# 

**Published:** 1891-02

**Authors:** 


### CONTENTS—FEBRUARY, 1891.—VOL. VI., NO. 8.

ORIGINAL ARTICLES— PAGE.

A Case of Extra-Uterine Pregnancy of Four Months, delivered per uterus. J. W. Carhart, M. D ............ 325

Small-Pox.—Vaccination. W. M. Yandell, M. D...... 330

Surgical Clinic. Memphis Hospital Medical College Service of W. B. Rogers, M. D., Prof. Surgery .......... 334

The Duty of Texas Legislators on Sanitation. O. Eastland, M. D .................................... 337

Plantar Neuritis; Resection of Plantar Nerve. T. J. Bennett, M. D ....................................... 341

SELECTIONS—

Carlsbad Water and the Sprudel Salt.................. 343

Hints in Gynecological Practice .................... 349

SOCIETY NOTES—

Panhandle Medical Society. Austin District Medical Society. U. S. Practitioners' Protective Alliance. Central Texas Medical Society. Circular Letter Texas Texas State Medical Association.................... 352

EDITORIAL—

Public Health, Public Wealth......................... 355

The Medical Bill. Dr. John Preston. Dr. W. W. Reeves.........................................359-61

MEDICAL NEWS AND MISCELLANY—

Numerous items on numerous and diverse subjects of interest to readers.......................................361–3

PUBLISHER'S NOTES—

Concerning our advertisers, all of interest to practicing physicians.......................................... 368

